# Characterization of the mitogenomes of long-tailed giant rat, *Leopoldamys sabanus* and a comparative analysis with other *Leopoldamys* species

**DOI:** 10.1080/23802359.2021.1872433

**Published:** 2021-02-11

**Authors:** Puteri Nur Syahzanani Jahari, Shahfiz Mohd Azman, Kaviarasu Munian, Nor Hazwani Ahmad Ruzman, Mohd Shahir Shamsir, Stine R. Richter, Faezah Mohd Salleh

**Affiliations:** a Department of Biosciences, Faculty of Science, Universiti Teknologi Malaysia, Johor, Johor Bahru, Malaysia; b Forest Biodiversity Division, Forest Research Institute Malaysia, Selangor, Kepong, Malaysia; c Faculty of Applied Sciences and Technology, Universiti Tun Hussein Onn Malaysia, Pagoh Higher Education Hub, Johor, Muar, Malaysia; d Section for Evolutionary Genomics, The GLOBE Institute, University of Copenhagen, Copenhagen, Denmark

**Keywords:** *Leopoldamys sabanus*, mitogenome, phylogenetic analysis, genetic connectivity

## Abstract

Two mitogenomes of long-tailed giant rat, *Leopoldamys sabanus* (Thomas, 1887), which belongs to the family Muridae were sequenced and assembled in this study. Both mitogenomes have a length of 15,973 bp and encode 13 protein-coding genes (PCGs), 22 transfer RNA genes, two ribosomal RNA genes and one control region. The circular molecule of *L. sabanus* has a typical vertebrate gene arrangement. Phylogenetic and BLASTn analysis using 10 *Leopoldamys* species mitogenomes revealed sequence variation occurred within species from different time zones. Along with the taxonomic issues, this suggests a landscape change might influence genetic connectivity.

The long-tailed giant rat, *Leopoldamys sabanus* is the common, generalist species in local assemblages of small mammals. This species is present throughout the Sunda region of Southeast Asia (Lim 1970) and has high mobility ranging between various forest matrices such as logged and unlogged forests (Wells et al. 2008). Recently, *L. sabanus* is reported to be widely distributed compared to the other non-volant mammals in northern forests of Peninsular Malaysia (Munian et al. 2020). In this study, we determined additional complete mitogenomes of *L. sabanus* from Malaysia that could be an important resource for addressing taxonomic issues and studying landscape genetics.

*Leopoldamys sabanus* sequenced in this study were collected from Bukit Tarek Forest Reserve, Selangor, Malaysia (3.48 N 101.47 E) (Faradiana et al. 2019) and Bukit Belate, Selangor, Malaysia (2.25 N 102.30 E). Total genomic DNA was extracted from the specimen tissues, which has been deposited at the Zoological Collection of Forest Research Institute Malaysia (FRIM) (Voucher No. MZF1958 and MZF731). The library was constructed using Blunt-End Single-Tube (BEST) protocol (Carøe et al. 2018). The mitogenome was assembled and annotated following Jahari, Abdul Malik, et al. (2020) and Jahari, Mohd Azman, et al. (2020). Both mitogenomes of *L. sabanus* (Genbank accession no. MT241668 and MT259591) have a length of 15,973 bp includes 13 protein-coding genes (PCGs), 22 transfer RNA genes, two ribosomal RNA genes and one control region.

These two *L. sabanus* mitogenomes display overall nucleotide composition which is 33.62% A, 28.68% T, 12.52% G and 25.17% C. The A + T content (62.30%) is higher than G + C content, which is similar to the other mitogenome of *Leopoldamys* species (Zhu et al. 2016; Camacho-Sanchez et al. 2017; Mohd Salleh et al. 2017). The total length of the protein-coding gene sequences (PCGs) is 11,405bp. The total length of the 22 tRNA genes is 1491bp, ranging from 57 bp (tRNASer) to 73 bp (tRNALeu). The 12S rRNA gene (955 bp) and 16S rRNA gene (1,573bp) are located between the tRNAPhe and tRNAVal, and between tRNAVal and tRNALeu, respectively. NAD6 and eight tRNAs genes (tRNAGln, tRNAAla, tRNAAsn, tRNACys, tRNATyr, tRNASer, tRNAGlu, tRNAPro) were encoded by the L-strand, other genes were encoded by the H-strand.

A phylogenetic tree of all available *Leopoldamys* mitogenomes was constructed using MEGA X software (Kumar et al. 2018). We confirmed that two *L. sabanus* in this study clustered with the other previously sequenced *L. sabanus* (Mohd Salleh et al. 2017; Nicolas et al. 2020) and rooted with the other *Leopoldamys* species (Zhu et al. 2016; Camacho-Sanchez et al. 2017) (Figure 1). The comparison of these two newly sequenced mitogenomes to the Genbank using BLASTn found the closest match (more than 98% similarity) to the same species. However, it also showed sequence variation (92% similarity) when matched to the other sample of the same species (Genbank accession no. KY117551) (Mohd Salleh et al. 2017). Particularly, sample KY117551, which is a historical specimen collected from Sarawak instead of Peninsular Malaysia and has nearly a 30 years gap with the other *L. sabanus* in this study. The vicariance event which occurred in the Miocene and early Pliocene in Sunda Shelf, landscape variation and time lag factors could possibly alter the genetic connectivity between certain terrestrial species including *Leopoldamys* species (Gorog et al. 2004; Spear and Storfer 2008; Waits et al. 2016). In addition, it is also worth considering that *L. sabanus* may represent a complex of cryptic species due to the same morphology (Musser and Carleton 2005; Tamrin and Abdullah 2011). Thus, the mitogenomes generated and the analyses provided in this study address not only the taxonomic issues of the *Leopoldamys* species but also suggest further investigation on landscape genetics to examine how landscape change could influence genetic connectivity within *Leopoldamys* species.

**Figure 1.**
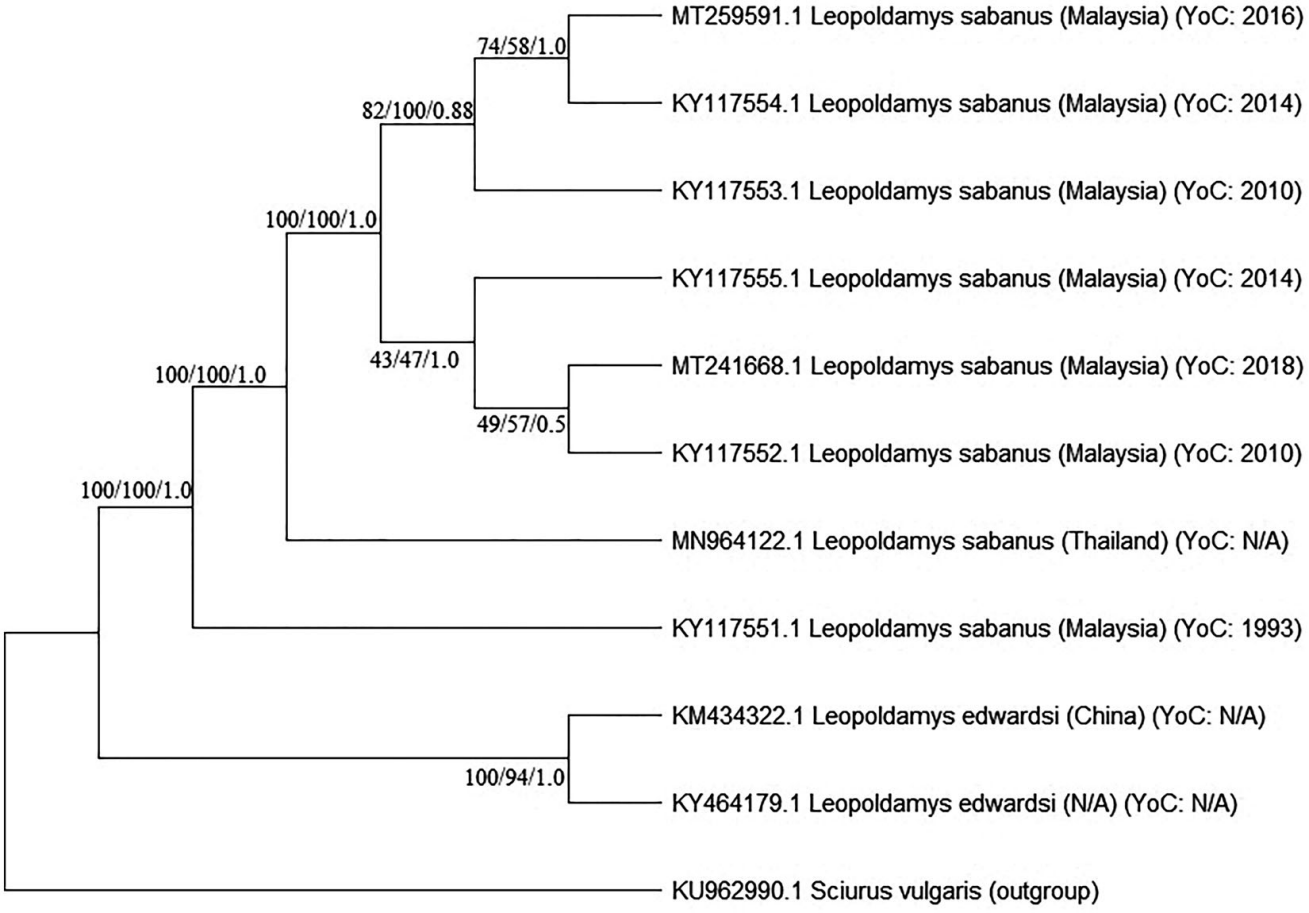
The phylogenetic tree of *L. sabanus* mitogenomes (MT241668 and MT259591) and other *Leopoldamys* species available in Genbank. Bootstrap values were indicated in each branch of the tree representing the result of NJ/ML/Bayesian probability. *Sciurus vulgaris* was selected as outgroup (NA: not available; YoC: Year of Collection).

## Data Availability

The genome sequence data that support the findings of this study are openly available in GenBank of NCBI at (https://www.ncbi.nlm.nih.gov/) under the accession no. MT241668 and MT259591. The associated BioProject, SRA, and Bio-Sample numbers are PRJNA610427, SRR11241207 and SRR11241244, SAMN14297804 and SAMN14297815, respectively.
